# Primary Segmental Small Bowel Volvulus in an Adolescent Female

**DOI:** 10.1055/s-0041-1735808

**Published:** 2021-11-24

**Authors:** Friederike Heidtmann, Felicitas Eckoldt, Hans-Joachim Mentzel, Ilmi Alhussami

**Affiliations:** 1 Department of Pediatric Surgery, Universitätsklinikum Jena, Klinik für Kinderchirurgie, Universitätsklinikum Jena, Jena, Germany; 2 Section of Pediatric Radiology, Institute of Diagnostic and Interventional Radiology I, Universitätsklinikum Jena, Jena, Thüringen, Germany

**Keywords:** abdominal pain, small bowel obstruction, segmental small bowel volvulus

## Abstract

Small bowel volvulus is a rare but important cause of abdominal pain and small bowel obstruction in children and adults. In the neonate, small bowel volvulus is a well-known complication of malrotation. Segmental small bowel volvulus is a lesser-known condition, which occurs in children and adults alike and can rapidly progress to bowel ischemia. Primary segmental small bowel volvulus occurs in the absence of rotational anomalies or other intraabdominal lesions and is rare in Europe and North America. Clinical presentation can be misleading, causing a delay in diagnosis and treatment, in which case the resection of necrotic bowel may become necessary.

We report on a 14-year-old girl who presented with severe colicky abdominal pain but showed no other signs of peritoneal irritation or bowel obstruction. An emergency magnetic resonance imaging was highly suspicious for small bowel volvulus. Emergency laparotomy revealed a 115 cm segment of strangulated distal ileum with no underlying pathology. We performed a detorsion of the affected bowel segment. Despite the initial markedly ischemic appearance of the affected bowel segment, the patient achieved full recovery without resection of bowel becoming necessary.

## Introduction

Small bowel volvulus (SBV) is defined as the torsion of the small bowel around its mesenteric axis. While midgut volvulus is a well-known complication of malrotation in neonates and young infants, segmental SBV may occur at any age. Primary and secondary segmental SBV have been differentiated. Secondary segmental SBV is caused by intraabdominal lesions like peritoneal adhesions, inflammatory processes, Meckel's diverticulum or other intraabdominal pathologies. 1 2 3 4 In contrast, primary segmental SBV occurs in a normal abdominal cavity without underlying rotational anomalies or other predisposing conditions. 4 While primary SBV appears to be a common cause of small bowel obstruction in children and adults in certain African and Asian countries, it is rare in Europe and North America. 3 Therefore, fairly little is known about the incidence, presentation, and management of SBV in western adolescents. This article reports on a case of segmental SBV in a 14-year-old girl.

## Case Report

A 14-year-old girl presented to the pediatric emergency department (ED) with severe abdominal pain. Two days before, an episode of intermittent, colicky abdominal pain had prompted her to present to the pediatric ED. Upon the first presentation, there was diffuse tenderness of the abdomen with no clinical signs of obstruction or peritonitis. She reported no vomiting and no fevers. Except for uncomplicated laparoscopic appendectomy 3 years earlier, there were no previous operations, no known chronic illnesses or allergies, and no significant family history. Her symptoms improved spontaneously upon evaluation by the pediatrician and were attributed to a nonurgent cause like nonspecific enteritis or mesenteric lymphadenitis, obstipation, functional pain, or a benign gynecologic condition. She was discharged and scheduled for a follow-up with abdominal ultrasound. At home, symptoms resolved further without analgesics, causing the patient to miss her scheduled follow-up the next day.

**Fig. 1:**
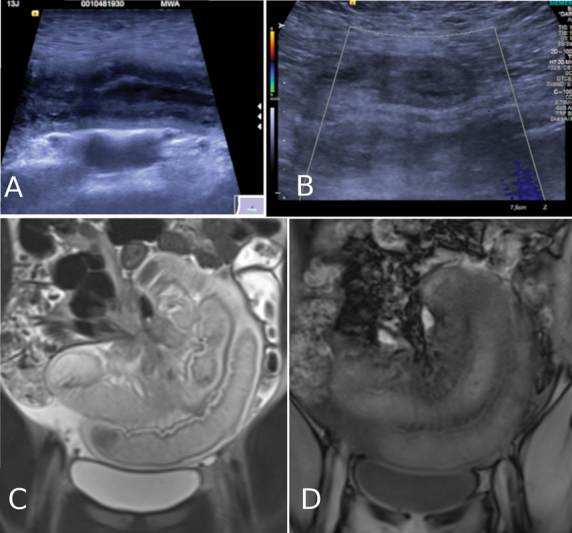
Notable on ultrasound is the appearance of dilated, thickened bowel loops ( **A** ) and a lack of perfusion on Doppler ultrasound ( **B** ). Abdominal magnetic resonance imaging demonstrated the torsion of bowel around the mesenteric pedicle (similar to a “whirlpool”-sign ( **C** ). There was no contrast medium uptake in the affected, thickened bowel segment ( **D** ).

**Fig. 2:**
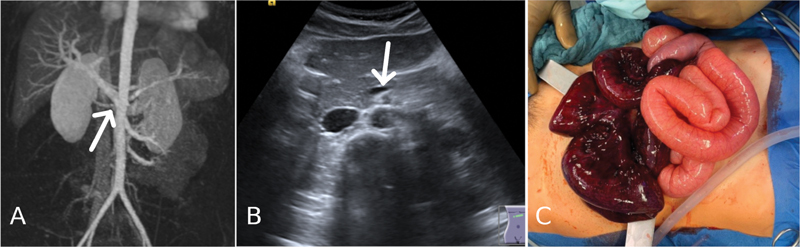
Contrast-enhanced magnetic resonance angiography demonstrated a slight ventralization of the venous confluence in front of the mesenteric artery (arrow) ( **A** ). B-mode ultrasound showed a slight clockwise ventralization of the superior mesenteric vein (arrow), which normally lies to the right of the superior mesenteric artery ( **B** ). Midline laparotomy revealed a 360 degrees-volvulus involving ∼115 cm of ileum up to 10 cm proximal of the ileocecal valve with marked intestinal ischemia. The cecum and colon were in their normal anatomical position (not shown) ( **C** ).

Two days later, the patient experienced a sudden onset of severe colicky abdominal pain. She presented to the pediatric ED by ambulance, having received analgesia with morphine and metamizole from the onsite emergency physician. She had not vomited, had had a bowel movement the morning of presentation with normal-appearing stool, and reported a normal appetite until the sudden onset of pain in the afternoon. She was evaluated by the on-call pediatrician, who ordered laboratory tests and abdominal ultrasound. Abdominal ultrasound showed marked distended small bowel loops in the lower quadrants with nonpropulsive peristalsis, thickened bowel wall, and blurred layers. There was no adequate perfusion in the wall using color Doppler imaging ( Fig. 1A , B ). Differential diagnosis after ultrasound included an intraabdominal inflammatory process leading to an obstruction of small bowel, while a strangulation of bowel, possibly due to intraabdominal adhesions, could not be ruled out. Laboratory results showed an elevated lactic acid (5 mmol/l) on peripheral venous blood gas analysis, while white blood cell count and C-reactive protein were in the normal range. The pediatric surgeon was called to the ED. On physical examination by the pediatric surgeon, the patient had a soft abdomen with diffuse tenderness in all quadrants. There were no guarding or other signs of peritoneal irritation. Bowel sounds were present. She was awake, cooperative, and hemodynamically stable. The remainder of the physical examination was unremarkable. Laboratory results did not show any sign of inflammation, while the presence of intraabdominal adhesions was not considered likely after uncomplicated laparoscopic appendectomy. An emergency contrast-enhanced magnetic resonance imaging (MRI) of the abdomen at 1.5 Tesla was performed to confirm the suspected diagnosis of bowel strangulation and evaluate for other intraabdominal and pelvic processes before urgently taking the patient to the operating room ( Fig. 1C , D ). It showed dilated, wall-thickened ileum loops in the lower abdomen, which did not show regular enhancement after contrast application (0.1 mmol/kg body weight macrocyclic gadolinium agent). Correlating MRI and ultrasound, it was noted that the venous confluence, which normally lies to the right of the superior mesenteric artery (SMA), appeared slightly anteriorized on ultrasound and MRI, but there was no diagnostic sign of malrotation or midgut volvulus on ultrasound ( Fig. 2A , B ). In conclusion, imaging was highly suspicious for segmental volvulus with rotation around the ileal vessels.

Midline laparotomy revealed a 360 degrees-volvulus of a 115 cm segment of the distal ileum, ending at 10 cm oral to Bauhin's valve ( Fig. 2C ). There were no adhesions and no signs of underlying anatomic pathology. After counterclockwise detorsion of the affected bowel, the profoundly ischemic ileum segment was placed in warm moist gauzes and observed for 20 minutes. Patchy reperfusion of the affected bowel was noted. In the absence of frankly necrotic bowel segments, no resection or stoma formation was performed. The bowel was placed back into the abdominal cavity and the abdomen closed.

The patient remained on parenteral nutrition for 5 days. After reintroducing oral fluids, one episode of bilious vomiting occurred and was managed nonsurgically with nasogastric tube decompression. Fortunately, the patient proceeded to recover well. She was discharged on the 12th postoperative day and is healthy today, with no recurrent symptoms 12 months later.

## Discussion

SBV describes the small intestine's clockwise torsion around its mesenteric axis that results in obstruction, ischemia, necrosis, or perforation of the affected bowel segment, with possible consequences like short-bowel-syndrome and death. 3 Unlike midgut volvulus secondary to malrotation, which is most common in the neonate and young infant, segmental SBV is known to occur at any age. 5 Annual incidence of SBV is reported to be 1.7 to 5.7 per 100,000 adults in Western countries, and 24 to 60 per 100,000 adults in Africa, the Middle East, and Asia. 3 4 Though primary SBV accounts for 31 to 100% of cases of SBV in case studies performed in certain Asian, African, and Middle Eastern countries, it is extremely rare in Europe and North America, accounting for an estimate of 0.1 to 0.22% of hospitalizations for bowel obstruction. 3 4 This difference in incidence is not fully understood, though it has been attributed to differences in dietary habits. 3 Information about segmental SBV in school-age children and adolescents is especially scarce and derived mostly from single case reports and small case series, which have usually featured children younger than 5 and older than 15 years. 3 6 7 8 9 10

Clinical diagnosis of segmental SBV is challenging due to its nonspecific presentation. 2 Whereas in neonates, abdominal distension, and bilious vomiting are common signs of midgut volvulus as well as segmental volvulus, signs of intestinal obstruction may be present but are not obligatory in older children and adults. 6 7 Abdominal pain is the earliest and most common symptom and may be colicky or steady. The onset of acute symptoms may be preceded by colicky epigastric pain several days before, as seen in this case. Abdominal pain that is disproportionally severe compared with the signs of obstruction may be a sign of ensuing vascular compromise. 2

Diagnostic imaging can support the preoperative diagnosis of SBV. Plain abdominal radiographs may show nonspecific signs of intestinal obstruction. 2 Ultrasound is sensitive (83–97.7%) and specific (84–100%) for recognizing small bowel obstruction, but has limitations and may not be able to identify the etiology of the obstruction. 11 Occasionally, a peripheral segmental vascular whirl suggestive of segmental strangulation may be observed on Doppler ultrasound. The diagnostic “whirlpool sign” in color Doppler ultrasound (wrapping of the superior mesenteric vein and the mesentery around the SMA) offers a high specificity for midgut volvulus, but as in our case, it is not usually present in segmental SBV. 6 12

In adult patients, abdominal computed tomography (CT) is the preferred imaging modality for small bowel obstruction (with sensitivities over 96% and specificities up to 100%) and suspected SBV, as it may show specific signs of volvulus (the “whirlpool” spiral appearance of the mesenteric vessels) as well as signs of obstruction and intestinal ischemia. MRI can provide conclusive evidence of volvulus without the risks of ionizing radiation and is an alternative to CT in children. 11 13 As seen in this case, it may be considered if the patient is stable and other forms of diagnostic imaging do not provide adequate information. Upper gastrointestinal series is used in stable patients to demonstrate malrotation but is not recommended in acutely ill patients with suspected strangulation ileus, where resuscitation and laparotomy should be performed emergently. 7 14 15 If clinical suspicion is high for acute bowel ischemia, further imaging studies should not delay surgical intervention.

Surgical treatment of primary segmental SBV aims at restoring the intestinal blood flow. Management following evisceration and detorsion depends mainly on the observed bowel viability. 6 In some cases, simple detorsion may suffice to recover an initially markedly ischemic bowel loop, while bowel that is frankly necrotic needs to be resected. The decision to perform a primary anastomosis versus a staged operation of segmental resection with ileostomy is not only an individual one but also depends on the patient̀s age at presentation. 6

Initial assessments of bowel viability based on clinical signs like discoloration of bowel and motility have shown to be imprecise. 16 In the presence of longer bowel segments of questionable viability, temporary closure and reevaluation in a second-look operation after 24 to 48 hours may allow for the preservation of as much bowel as possible. 17 Second-look laparotomy has been successfully applied in neonates with midgut volvulus and malrotation. 18 Its role in SBV beyond the neonatal and infant period is less established, although it is an accepted course of treatment for acute mesenteric ischemia in adults. 16

While mortality is raised significantly in the presence of gangrenous bowel, there is potential for patients to recover uneventfully after detorsion of SBV without bowel resection. 2 3 4 9 19 20 In patients who appear clinically well after the first exploration, it seems reasonable to take a relaparotomy on-demand approach. In our case, a markedly ischemic-appearing bowel segment fully recovered while sparing the patient the additional risks and complications of bowel resection or a second surgery.

## Outlook

SBV should be considered in a child presenting with a sudden onset of severe abdominal pain and vomiting. Management after emergency laparotomy and detorsion is individual and depends on the observed bowel viability. Early diagnosis and treatment are crucial to avoid complications like bowel infarction with loss of significant amounts of short bowel as well as a raised overall mortality.

## Notes

New Insights and the Importance for the Pediatric Surgeon Primary small bowel volvulus is rare and clinical presentation can be misleading. Early diagnosis and treatment are necessary to avoid bowel necrosis and resulting complications like malabsorption, short-bowel syndrome, and death.
